# Non-Canonical Regulation of Type I Collagen through Promoter Binding of SOX2 and Its Contribution to Ameliorating Pulmonary Fibrosis by Butylidenephthalide

**DOI:** 10.3390/ijms19103024

**Published:** 2018-10-04

**Authors:** Hong-Meng Chuang, Li-Ing Ho, Mao-Hsuan Huang, Kun-Lun Huang, Tzyy-Wen Chiou, Shinn-Zong Lin, Hong-Lin Su, Horng-Jyh Harn

**Affiliations:** 1 Buddhist Tzu Chi Bioinnovation Center, Tzu Chi Foundation, Hualien 970, Taiwan; kavin273@gmail.com (H.-M.C.); spleo0825@gmail.com (M.-H.H.); shinnzong@yahoo.com.tw (S.-Z.L.); 2 Department of Life Sciences, Agricultural Biotechnology Center, National Chung Hsing University, Taichung 402, Taiwan; 3 Division of Respiratory Therapy, Department of Chest Medicine, Taipei Veterans General Hospital, Taipei 112, Taiwan; breathho@gmail.com; 4 Hyperbaric Oxygen Therapy Center, Division of Pulmonary and Critical Care Medicine, Graduate Institute of Aerospace and Undersea Medicine, Department of Internal Medicine, Tri-Service General Hospital, National Defense Medical Center, Taipei 114, Taiwan; kun@mail.ndmctsgh.edu.tw; 5 Department of Life Science and Graduate Institute of Biotechnology, National Dong Hwa University, Hualien 974, Taiwan; twchiou@mail.ndhu.edu.tw; 6 Department of Neurosurgery, Buddhist Tzu Chi General Hospital, Tzu Chi University, Hualien 970, Taiwan; 7 Department of Pathology, Buddhist Tzu Chi General Hospital, Tzu Chi University, Hualien 970, Taiwan

**Keywords:** pulmonary fibrosis, butylidenephthalide, SOX2, type I collagen, bleomycin

## Abstract

Pulmonary fibrosis is a fatal respiratory disease that gradually leads to dyspnea, mainly accompanied by excessive collagen production in the fibroblast and myofibroblast through mechanisms such as abnormal alveolar epithelial cells remodeling and stimulation of the extracellular matrix (ECM). Our results show that a small molecule, butylidenephthalide (BP), reduces type I collagen (COL1) expression in Transforming Growth Factor beta (TGF-β)-induced lung fibroblast without altering downstream pathways of TGF-β, such as Smad phosphorylation. Treatment of BP also reduces the expression of transcription factor Sex Determining Region Y-box 2 (SOX2), and the ectopic expression of SOX2 overcomes the inhibitory actions of BP on COL1 expression. We also found that serial deletion of the SOX2 binding site on 3′COL1 promoter results in a marked reduction in luciferase activity. Moreover, chromatin immunoprecipitation, which was found on the SOX2 binding site of the COL1 promoter, decreases in BP-treated cells. In an in vivo study using a bleomycin-induced pulmonary fibrosis C57BL/6 mice model, mice treated with BP displayed reduced lung fibrosis and collagen deposition, recovering in their pulmonary ventilation function. The reduction of SOX2 expression in BP-treated lung tissues is consistent with our findings in the fibroblast. This is the first report that reveals a non-canonical regulation of COL1 promoter via SOX2 binding, and contributes to the amelioration of pulmonary fibrosis by BP treatment.

## 1. Introduction

Pulmonary fibrosis is a disease that occurs when scar-like tissue accumulates extracellular matrix (ECM) components (such as collagen, elastin, and fibronectin) in the pulmonary interstitial tissue. Such prolonged fibrogenesis makes the pulmonary tissue lose elasticity, resulting in a gradual loss of ability to contract, relax, and exchange gas. Statistics show that patients with idiopathic pulmonary fibrosis (IPF) have a two- to three- year median duration of survival from the time of diagnosis [1]. When definitively diagnosed, most patients’ pulmonary functions have already deteriorated, showing symptoms of dyspnea, leading to chronic hypoxia [2]. The efficacy of current medications is not commensurate with other treatments such as pulmonary transplantation. For example, despite clinical data showing little efficacy with anti-inflammatory agents, inflammatory effects after injury play an important role in fibrogenesis. However, the biggest disadvantage of pulmonary transplantation is the time it takes to find a suitable transplant. Thus, this unmet medical need requires the development of new targets [3].

In a classical model of fibrosis, TGF-β pathways play a pivotal role [4] in inducing (1) secretion of growth factors that facilitate the proliferation of connective tissues [5]; (2) epithelial-mesenchymal transition (EMT) in alveolar type I or alveolar type II cells such as a myofibroblast source [6]; and (3) regulation of metalloproteinase activity which results in tissue remodeling [7]. Inhibition of TGF-β using approved clinical treatments for IPF (Pirfenidone) causes common side effects such as nausea and photosensitivity [8]. In TGF-β signaling, receptor phosphorylates JNK, Akt, and p38 regulate cell proliferation and differentiation, affecting downstream pathways like Smad and non-Smad pathways. Phosphorylation of Smad2/3 regulates ECM deposition and EMT induction, whereas, the non-Smad pathway improves the proliferation of fibroblast [9]. A typical signaling pathway in TGF-β enhances the expression of collagen and other fibrogenic genes, and collagen deposition is the most common pathological finding in patients with pulmonary fibrosis. Fibrillar-type collagens, including type I, II, III, and V, also express during tissue fibrosis [10], with collagen I predominating in areas of mature fibrosis [11].

There has been evidence indicating the requirement of SOX2 expression in induced skin fibrosis, recruited into fibrotic lesions in response to bleomycin treatments [12]. There is also evidence of elevated SOX2 expression in IPF patients [13]. As such, we postulate a possibility of the requirement of SOX2 in pulmonary fibrosis. SOX2 exhibits pluripotent qualities, allowing cells to reprogram, but also induces tumorgenecity. In general, SOX2 functions and pathways are too complicated, making it difficult to explain its relationship with fibrogenesis. One possibility is that the specific promoter consensus sequences of SOX2, reported previously, drive expressions such as fibroblast growth factor 4 (*Fgf4*) [14]. Another author suggests that in bronchiolar Clara cells, SOX2 forms a complex with Smad3 and influences cell proliferation [15]. Nevertheless, these studies focus on the importance of SOX2 but not on the regulatory activity of its transcription. To address this, we have found in our previous studies that Butylidenephthalide (BP), also known as (3E)-3-butylidene-2-benzofuran-1-one in IUPAC, showed anti-fibrotic effects in the liver, with its mechanism enhancing Bone morphogenetic protein 7 (BMP7) expression and reducing TGF-β in hepatic stellate cells (HSC) [16]. The HSC functions as a fibroblast after EMT, and can be reduced through BP treatment. In addition, BP decreases the expression of SOX2 by reducing its metastasis and invasiveness in glioblastoma multiform (GBM) cell [17].

Since our previous study demonstrated the effect of BP in treating liver fibrosis, it is possible that BP is effective for pulmonary fibrosis. To this end, we sought to examine the anti-fibrotic effect of BP on pulmonary fibrosis. Our data indicates that while BP reduced the expression of Collagen I, it altered neither the TGF-β-induced downstream pathways, nor the EMT status, and the expression of Collagen I was recovered in the exogenous overexpression of SOX2 in lung fibroblasts. Moreover, a consensus the SOX2 binding region was found in the promoter region of *COL1A1*, where BP reduction of SOX2 and its binding activity on the collagen I promoter leads to the inhibition of collagen deposition. The relationship between SOX2 and *COL1A1* was further examined by using chromatin immunoprecipitation. In the bleomycin-induced mouse model, treatment groups were found to not only ameliorate the fibrosis score, but also the pulmonary function. All in all, these findings reveal a BP-induced non-canonical regulation of SOX2, and a potential candidate for the amelioration of pulmonary fibrosis.

## 2. Results

### 2.1. BP Treatment in Human Lung Fibroblasts Attenuated Collagen Expression Driven by Exogenous TGF-β

**Figure 1:**
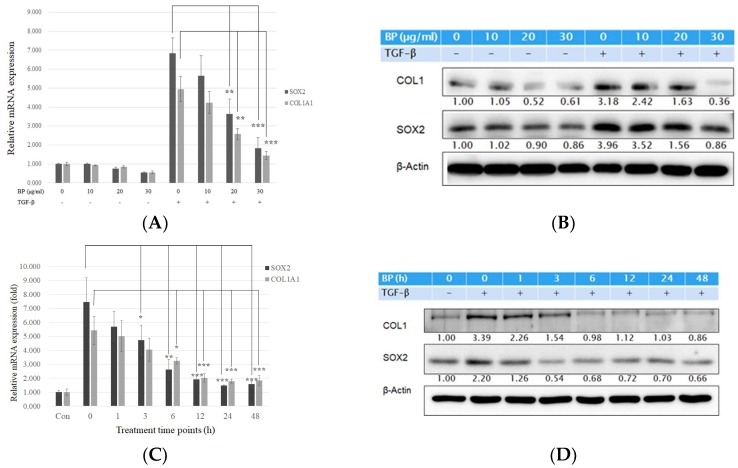
BP reduced collagen I production in lung fibroblast pre-stimulated with recombinant TGF-β1. TGF-β (5 ng/mL) stimulate for 12 h and (**A**) mRNA and (**B**) protein expression levels of BP treatment in several dosages (0, 10, 20, and 30 µg/mL) for 24 h on type I collagen, and SOX2 expressions. (**C**) mRNA and (**D**) protein expression levels of BP treatment for several time points (0, 1, 3, 6, 12, 24, and 48 h) in the dosage of 30 µg/mL on type I collagen and SOX2 expressions. The same amount of DMSO was added in 0 µg/mL groups as a vehicle control. Data showed 3 independent qPCR experiments and presented are mean ± SD. * denotes a significant decrease with the 0 µg/ml group of *p* < 0.05; ** *p* < 0.01; *** *p* < 0.001 by student’s *t*-test.

The most extensive evidence on the origin of the myofibroblast is from fibroblast transdifferentiation. Intensive studies have shown that TGF-β1 would induce a similar progression of pulmonary fibrosis in mice [18], where treatment of TGF-β1 in isolated lung fibroblasts induces fibrotic reaction such as collagen and cytokines expression [19]. To evaluate the effects of BP in lung fibrosis, we established an in vitro model using normal human lung fibroblast cell lines (NHLF). Previous studies have shown that TGF-β1 is an effective cytokine to induce various types of collagen [20]. After stimulating with exogenous TGF-β treatment, cells were treated with or without BP (0 to 30 µg/mL) for 24 h. As shown in Figure 1A, the mRNA levels of type I collagen α (*COL1A1*) were dramatically increased by TGF-β (1.0 vs. 4.9), but decreased in BP treatment groups. In addition, BP reduces mRNA and protein levels of type I collagen in a time-dependent manner (Figure 1C,D). The protein and mRNA levels in SOX2 were found to have similar trends (response to dosage) as those of type I collagen (Figure 1A,B). These results suggest that BP could reduce collagen production, and that it has a potential for treating lung fibrosis.

### 2.2. BP Did Not Regulate the Smad and Non-Smad Pathways to Block the Effects of TGF-β

**Figure 2:**
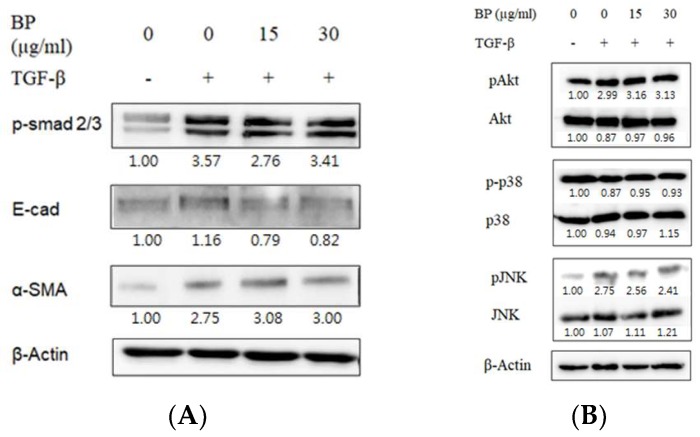
Effect of BP in canonical TGF-β downstream Smad and non-Smad pathways. Representative images of western blot analyses for TGF-β-induced (**A**) phosphorylated Smad2/3 for Smad pathway and its downstream EMT markers, and (**B**) phosphorylated Akt, p38 and JNK for non-Smad pathways and the effect of BP treatment.

Previous reports have concluded that the role of TGF-β through the phosphorylation of Smad2/3 leads to the stimulation of ECM production in pulmonary fibroblasts [21]. In this study, TGF-β1 stimulation in normal lung fibroblast cell lines (NHLF) successfully upregulated in the expression of collagen and phosphorylation of Smad 2/3. Remarkably, BP did not reduce the phosphorylation of the downstream messenger of TGF-β, Smad2/3 (Figure 2A). Previous studies suggest that TGF-β-induced EMT results in the loss of E-cadherin expression, breaking the tight junctions in the epithelial type cells. However, in this study, the expression of E-cadherin was not changed by BP, as shown in Figure 2A, suggesting that BP would not alter the EMT status in lung fibroblasts. In this study, the NHLF treated TGF-β1 was sufficient to induce non-Smad pathways such as JNK, Akt, and p38 phosphorylation (Figure 2B). This treatment did not reduce the phosphorylation in these pathways, suggesting that BP would not alter the canonic TGF-β signal pathways. 

### 2.3. SOX2-Overexpression Prevented Collagen Reduction in BP Treatment

**Figure 3:**
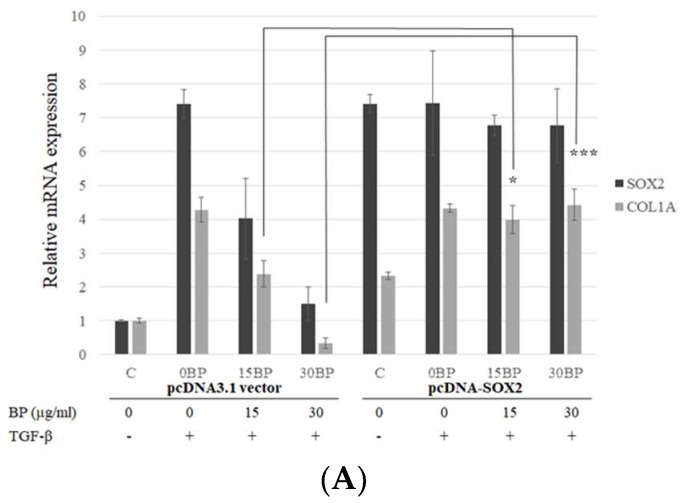

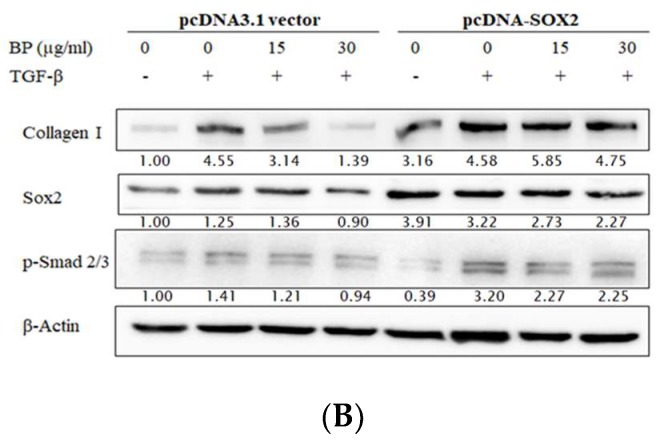
Effects of exogenous overexpression of SOX2 on BP treatments in fibroblasts. Stable expression lines of pcDNA3.1/SOX2 and its vector control were isolated by G418 and treated with or without BP for 24 h. (**A**) Analysis of the Col I and SOX2 expressions were done on the mRNA levels by qPCR; and (**B**) analysis of the Col I, phospho-Smad2/3, and SOX2 expressions were done on the protein level by Western blot analysis (representative images). Data showed 3 independent qPCR experiments and presented are mean ± SD. * denotes a significant decrease with the - group of *p* < 0.05; *** *p* < 0.001 by student’s *t*-test.

Although SOX2 plays an important role in fibroblast differentiation, the relationship between SOX2 and fibrosis is still unclear. Weina et al. proposed that SOX2 might be involved in the TGF-β signaling pathway and correlates it with melanoma aggressiveness and metastasis in human melanoma cells [22]. Our previous study has shown that BP reduced SOX2 expression and stemness in glioblastoma cancer stem cells [17]. In this study, SOX2 expression was reduced in a time-and dose-dependent manner in BP-treated NHLFs. In order to reveal the relationship between SOX2 and BP-reduced collagen production, we constructed a SOX2 coding sequence on the pcDNA3.1 expression vector. NHLF transfected pcDNA3.1/SOX2 increased mRNA (Figure 3A) and protein (Figure 3B) levels of SOX2. However, in SOX2-overexpressed cells, BP treatment did not decrease type I collagen in both mRNA and protein levels, while the vector control group still decreased SOX2 and collagen. These finding suggested that the expression of collagen might be correlated by SOX2.

### 2.4. BP Decreased SOX2 Specific Promoter Binding on Collagen

**Figure 4:**
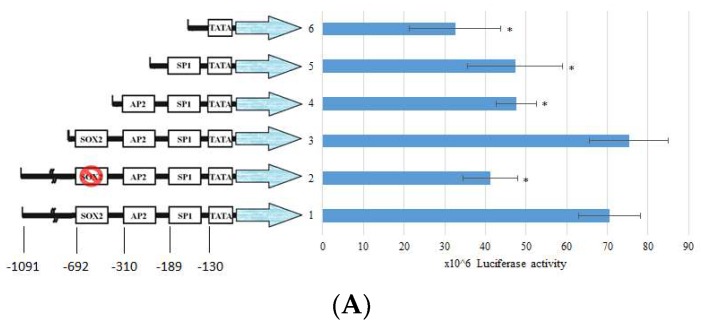

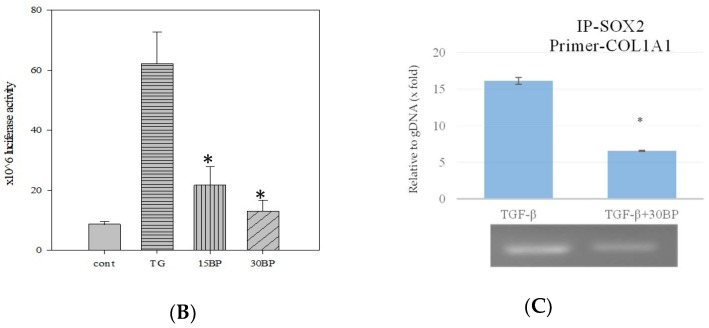
Effects of BP and SOX2 on the activity of type I collagen promoter deletion constructs in NHLF. Luciferase reporter gene constructs including truncated sequences of the *COL1A1* promoter and (**A**) Responsive elements exhibiting the previous described localization of consensus motifs of transcription factors SOX2, AP2, SP1, and TATA box are indicated. (**B**) The luciferase activity was determined 45 h after transfection of pGL3/COL1A1 in NHLF cells and BP treatment. Numbers represent the mean of 4 independent experiments. (**C**) ChIP analysis of SOX2 binding on the full length *COL1A1* promoter in BP-treated groups, and CT values (upper) were obtained from qPCR analysis and the representative image (bottom) from three independent experiments of gel electrophoresis, respectively. * denotes a significant decrease with the full-length group of *p* < 0.05 by ANOVA followed by *post hoc* analysis of Dunnett’s test.

The regulation of collagen expression has been extensively studied, and components such as SP1 and AP2 contributing to its activity are already known. However, the consensus binding of SOX2 has still not been elucidated. To further elucidate whether the expression of collagen is reduced by BP, we constructed the type I collagen promoter sequence from −1091 to +3 of transcription starting site (TSS), as described in a previous report [23]. According to previous reports [24], the *COL1A1* promoter has a consensus SOX2 binding sequence, (T/A)TTGTT. Thus, we divided the promoter sequence into (1) a full length of *COL1A1* promoter; (2) a SOX2 binding site mutant construct; (3) a SOX2-AP2-SP1 binding site containing promoter; (4) a AP2-SP1 binding site containing promoter; (5) a SP1 binding site containing promoter; and (6) a TATA box only promoter (Figure 4A). The luciferase activity showed a significant decrease in both SOX2 binding site mutant and removal promoters, suggesting that the consensus SOX2 binding sequence, (T/A)TTGTT, could regulate the expression of *COL1A1*. To address whether BP might reduce the expression through the promoter regulation, we evaluated the luciferase activity in the condition of BP treatment (Figure 4B). The elevated luciferase activity in TGF-β-induced cells is significantly reduced by BP treatment (15, 30 µg/mL), and there was no difference between the control and BP treatment groups. To further define the binding characteristics of SOX2, we used chromatin immunoprecipitation to isolate the specific binding of SOX2 of the promoter sequence in TGF-β-induced NHLF cells. We found the consensus SOX2 binding site, (T/A)TTGTT, containing *COL1A1* promoter existed in the anti-SOX2 bound amplicons. In the result of qPCR analysis, SOX2 binding amplicon was markedly decreased while NHLF was being treated by BP (16.12 vs. 6.56) (Figure 4C). These finding suggest that the expression of type I collagen could regulated by the promoter-binding ability of SOX2 in response to BP treatment.

### 2.5. BP Reduced bleomycin-Induced Pulmonary Fibrosis in Mice

**Figure 5:**
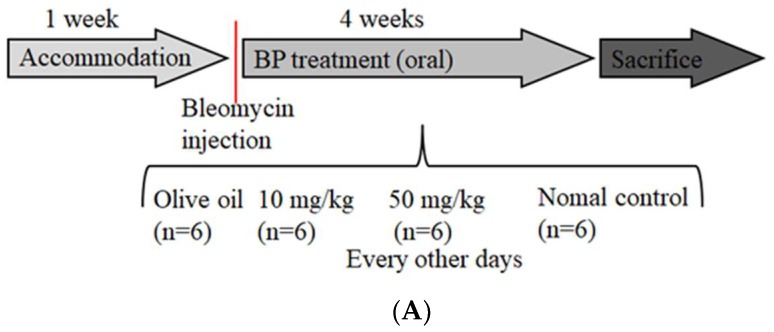

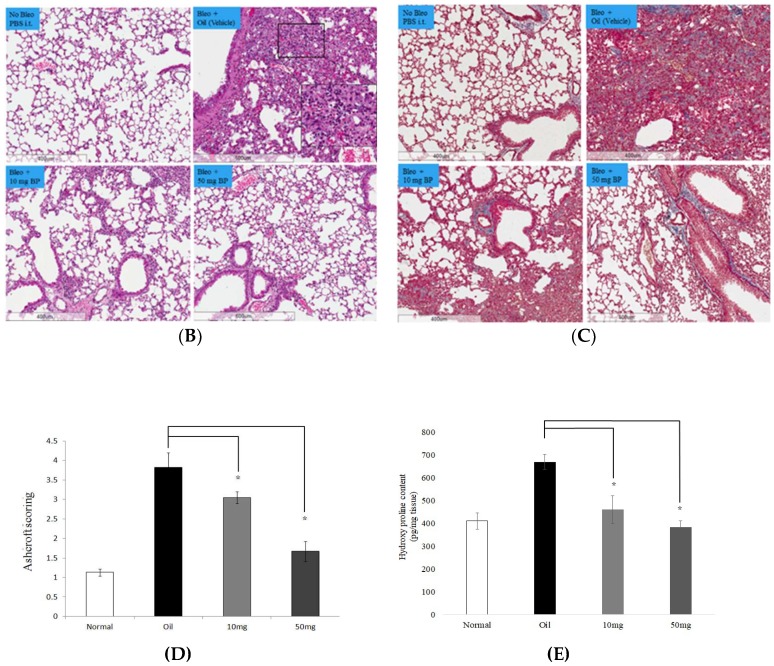
BP treatment in bleomycin-induced mice. (**A**) Schematic illustration experimental design. Mice were divided into four groups, and bleomycin was instilled on day 0 in all three experimental groups. The control group instilled the same volume of saline. Treatment groups were administered 0 (vehicle), 50 and 10 mg/kg doses of BP every other day for 30 days. Histological findings revealed lung inflammation and fibrosis by both (**B**) H&E (200 × magnification) and (**C**) Masson’s staining (100× magnification). (**D**) The Ashcroft score was measured with all field of slides, each view counted and represented with mean ± SEM from six mice per group. (**E**) The hydroxyl proline content was obtained from tissues of each lobes of lung. * denotes a significant decrease of *p* < 0.05 by ANOVA followed by post hoc analysis of Dunnett’s test.

To determine whether BP regulates the fibrotic phenotype in vivo, we delivered BP with oral administration to bleomycin-treated mice of lung injury (Figure 5A). BP treatment of C57B/L6 mice did not result in weight loss and death at both high (50 mg/kg) and low (10 mg/kg) dosages (data not shown). In terms of histology analysis, the increased cell infiltration in bleomycin-induced groups was reduced by dose-dependent BP treatment (Figure 5B). The enlarged figure (shown in the vehicle group) demonstrates a noted increase of inflammatory cells according to their morphology. The collagen deposition was indicated in the blue color of the tissues by Masson-trichrome staining, and more severe fibrosis in the vehicle group mice was observed as compared to that in BP treated groups (Figure 5C). Based on the lung thickening and the distortion structure of the histological feature, the Ashcroft score was found to significantly reduced in 10 mg/kg (*p* < 0.05) and 50 mg/kg (*p* < 0.05) of BP treatment groups (Figure 5D).

**Figure 6:**
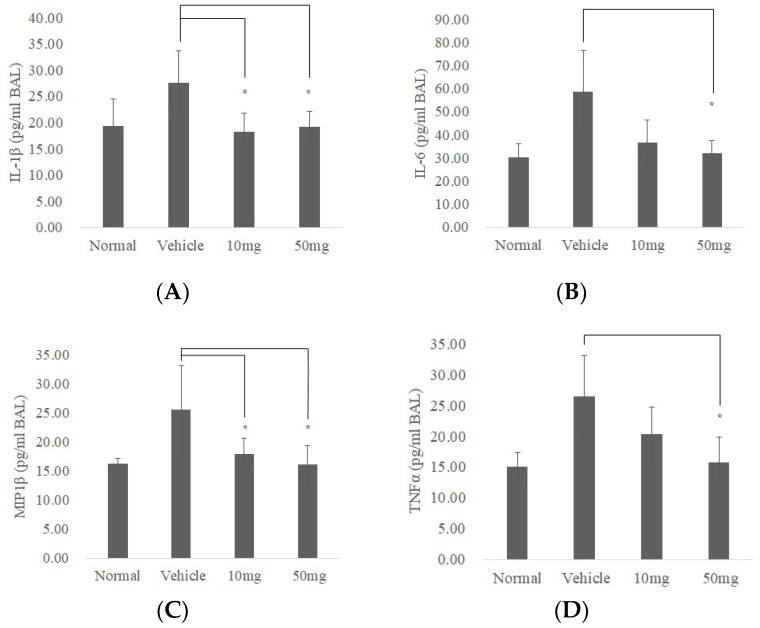

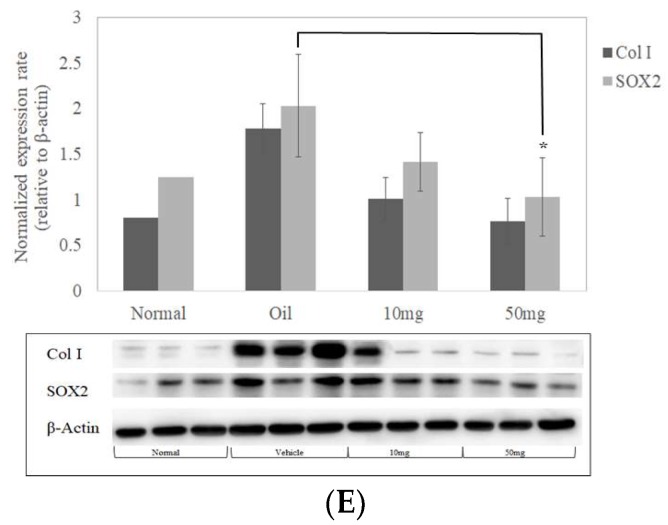
BP reduces inflammatory response and expressions of collagen I and SOX2 in bleomycin-induced mice. BALF was isolated (*n* = 5) and analyzed by ELISA for changes in (**A**) IL-1β, (**B**) IL-6, (**C**) MIP1β, and (**D**) TNF-α. (**E**) Expressions of collagen I and SOX2 protein from lung tissues in mice (*n* = 3) were quantified by Western blot and standardized by β-Actin. * denotes a significant decrease of *p* < 0.05 by ANOVA followed by *post hoc* analysis of Dunnett’s test. Data presented are mean ± SD. (BALF, broncho alveolar lavage fluid; MIP-1β, Macrophage Inflammatory Proteins 1 beta.).

Data from BAL fluid investigation showed IL-1β, IL-6, Macrophage inflammatory protein-1β (MIP-1β), and TNFα (which was reported as pulmonary fibrosis-related cytokines in BAL [25]) significantly decreased in BP treatment groups (Figure 6A–D. *p* < 0.05). These results suggest that BP is effective in ameliorating pulmonary fibrosis in bleomycin-induced mice mediated both by reducing collagen fiber and decreasing fibrotic related cytokines.

### 2.6. BP Restored Pulmonary Function in Bleomycin-Treated Mice

Bleomycin-induced pulmonary fibrosis is one of the most convincing animal models for investigating potential therapies, as its progression is consistent with patients who have developed lung fibrosis from bleomycin treatment. In our study, we administered bleomycin intratracheally to observe lung histopathology and pulmonary functions. In the treatment groups, we performed two doses of BP (50 and 10 mg/kg), and used olive oil as a vehicle control. On day 30, pulmonary functions were measured by whole body plethysmography. The respiratory rate and tidal volumes did not change in each of the groups. However, the Penh, EEP, relaxation times, and minute volume all significantly decreased (*p* < 0.05) (Table 1.) in BP treatment groups compared to the vehicle group and even the control group. Penh is used as an empirical index of airway resistance [26], and the result suggests that lung stiffness decreased and airway functions improved after BP treatment.

### 2.7. SOX2 and Collagen Expression Reduced in BP-Treated Lung Tissues

To further examine the inhibitory effects of SOX2 in BP-treated mice, we extracted protein lysate from lung tissues and performed western blot analysis. Protein expressions were quantified using immunoblot and its histogram plot show that both SOX2 and Collagen I expressions were markedly reduced in 50 mg/kg (Figure 6E).

## 3. Discussion

Our findings show evidence of BP as a potential therapeutic treatment in pulmonary fibrosis. Because fibroblasts and/or myofibroblasts in the fibrotic foci respond to the collagen synthesis [27], lung fibroblast cells are used to identify the inhibitory effects of fibrosis. From dose- and time-dependent studies, BP reduces the expression of collagen I in TGF-β1-treated NHLF. However, BP treatment did not alter Smad phosphorylation, nor non-Smad pathways such as the JNK, p38, and Akt. Interestingly, our previous data showed that SOX2 correlates with stemness but not with EMT status [17]. TGF-β signaling also induces SOX2 expression, reiterating the relationship between SOX2 and fibrogenesis. Thus, we conducted an ectopic expression of SOX2 to reactivate the collagen expression in BP-treated groups. In summary, these findings revealed that without the inhibition of the canonical TGF-β signaling pathways, BP decreases collagen deposition via SOX2 regulation.

In previous studies, ChIP-sequence analysis showed that binding activity of SOX2 on *COL1A1* promoter decreases when embryonic stem cells (ESC) differentiates into neural progenitor cells (NPC) [28], leading to a reduction in cell development with fibroblast characteristics. These findings suggest that direct regulation of the SOX family proteins is essential for collagen expression, as well as fibrosis. Unfortunately, these studies didn’t indicate the specific binding motif. Hence, we constructed a series of promoter deletions in the current study and found reduced reporter activity in the −722~+3 region in the promoter, removing a SOX2 consensus binding site. Furthermore, the promoter activity was significantly reduced in BP-treated TGF-β-induced NHLF (Figure 4B). Collectively, BP reduces the expression of SOX2 and Collagen I (Figure 1A), and regulates *COL1A1* promoter activity through SOX2 binding site (Figure 4A), suggesting a relationship in the regulation of SOX2 and *COL1A1*. In ChIP assay, direct binding of SOX2 was found in the *COL1A1* promoter region (Figure 4C). Inhibition of collagen synthesis is able to prevent bleomycin-induced fibrosis in vivo by not only restriction of serine/glycine uptake, but also through the key serine and glycine synthesis enzyme, phosphoglycerate dehydrogenase [29]. This recent study suggested that TGF-β-induced collagen synthesis and bleomycin-induced pulmonary fibrosis could inhibit fibrogenesis through collagen protein synthesis. Similarly, our result showed that a collagen transcription regulation is a potential therapeutic target; however, blocking serine and glycine uptake may influence other translation levels. Based on these findings, our study showed that BP can reduce collagen production and fibrogenesis potentially through SOX2 binding cis-element.

To further confirm our hypothesis of fibrosis reduction through BP, we established a bleomycin-induced pulmonary fibrosis animal model. The bleomycin-induced mice produced excessive collagen in our vehicle control groups, measured using Masson-trichrome staining (Figure 5C) and hydroxyl proline content assay (Figure 5E). The excessive collagen can be repressed with BP treatment, which is consistent with our results in human lung fibroblasts (Figure 1). The change in histopathology also shows more alveoli for better air exchange (Figure 5B), such that the accumulated and minute volumes are significantly restored in BP treatment groups (Table 1). Increased pulmonary fibrosis positive correlates with enhanced pause (Penh) [30,31], and our data showed a significant reduction in Penh compared to vehicle control groups, suggesting restoration of the respiratory function with a non-invasive measurement. Although our current study conducts a SOX2 knockout mice in animal models, the expression of SOX2 still reduces in BP-treated lung tissues (Figure 6E), which supports our findings in the regulation of SOX2 and collagen, thus clarifying the therapeutic effects of BP in lung fibrosis.

Our data showed immune modulation not only in the lung tissues through infiltration of multinucleated giant cells (Figure 5B, bleomycin with vehicle treatment), but also in the findings of BAL fluid (Figure 6), such as the IL-6 and IL-1β, that were both decreased in BP-treated groups. Other studies show that evoked T-helper cell 2 (Th2) is related to the excessive migration of macrophages and fibroblasts and resulted in Macrophage Inflammatory Proteins 1(MIP1) augmentation [32], as well as pulmonary fibrosis, further supporting our data [33]. Furthermore, our study suggests a possible relationship between inflammation and fibrosis, but the cause/result could not be determined in the current study. This might be a possible reason for the inefficient effects of immune-repressive drugs in the treatment of idiopathic pulmonary fibrosis patients [34].

**Figure 7:**
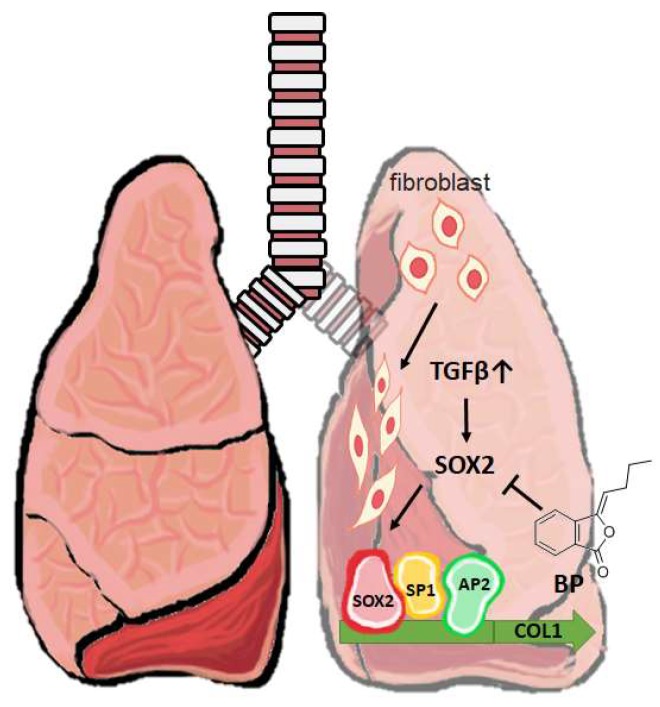
A short schematic mechanism of BP to treat pulmonary fibrosis. In a mouse model of bleomycin-induced pulmonary fibrosis, promoter binding enhances the collagen expression in fibroblast. BP inhibits SOX2 expression and its binding on collagen promoter blocks fibrogenesis in vitro and in vivo. ⊥: Inhibition of the gene expression; Gray upwards arrow ↑: Increased level of TGF-β.

Compared to our previous studies, a consistent down regulation in SOX2 was also observed in glioblastoma stem cell [17]. Chiou et al. reported a DNA methyltransferase 1 (DNMT-1) dependent manner which inhibits SOX2 expression through high-mobility group AT-hook 2 (HMGA2) and miR142-3p [35], where interleukin 6 (IL-6) induces DNMT-1 hypermethylation on the promotor of miR142-3p. Our previous microarray data in GBM observed a downregulation of *HMGA2* and *COL1A1* as well, suggesting that BP reduces these targets in different cell types. One possible explanation might be epigenetic modification, such as methylation or miRNAs. Moreover, the present findings in BAL fluid showed a reduction of IL-6 compared to the vehicle treatment group (Figure 6). Detailed mechanisms about BP-influenced regulation of SOX2 and its relationship between IL-6 and other cytokines are still unknown, and will be studied in the future. In summary, our present data supports SOX2 regulation of *COL1A1* promoter as a potential target in BP treatment of pulmonary fibrosis (Figure 7). 

## 4. Material and Methods

### 4.1. Chemical and Treatment

Butylidenephthalide (MW: 188.23), purchased from Lancaster Synthesis Ltd. (Newgate, Morecambe, UK), was dissolved in dimethylsulfoxide (DMSO), as previously described [36] for treatments in cell cultures. The same amount of DMSO was added as a vehicle control. For the animal study, BP was dissolved in food grade olive oil (Forlì, Italy). In vitro studies of lung fibrosis were performed in normal Human lung fibroblast (NHLF), which was stimulated into fibrogenesis by recombinant human TGF-β1 (PeproTech, Catalog Number: 100-21, 5 ng/mL) for 12 h.

### 4.2. Cell Culture and Transfection

NHLF cell was purchased from Lonza (Basel, Switzerland) and maintained in DMEM supplemented with bFGF (Peprotech, London, UK) (5 ng/mL), insulin (5 µg/mL), and 2% FBS (HyClone, Logan, UT, USA). Serum supplement was removed in TGF-β treatment because the presence of serum is likely to diminish the fibrogenic actions of TGF-β. The construct of pcDNA3.1/SOX2 was previously described [17], and NHLF was transfected with pcDNA3.1 vector or pcDNA3.1/SOX2 by FuGENE HD^®^ Transfection Reagent (Promega, Mannheim, Germany). Cells were then exposed to 600 μg/mL G418 (Invitrogen, Carlsbad, CA, USA) in a complete medium containing 2% FBS for stable clone selection over 3 weeks. The transfection efficiency was measured by Western blot for His-tag expression before the treatment of TGF-β and BP.

### 4.3. RT-PCR and Western Blot Analysis

After the treatment of BP, total RNA was isolated by RNeasy RNA isolation kit (Qiagen, Germantown, MD, USA). The subsequent RNA was quantified into 2 µg samples, and reverse transcription was performed by QuantiTect Reverse Transcription Kit (Qiagen, Germantown, MD, USA). The qPCR reactions were performed by the LightCycler^®^ SYBR Green I Master (Roche, Basel, Switzerland) reagent, and primers are listed in Tab S1. The semi-quantitative RT-PCR was performed according to the following program: 10 min 95 °C followed by 40 cycles of 15 s for 95 °C, 20 s for 60 °C, and 20 s for 72 °C, and followed by 5 min for 72 °C. The qPCR detection program was as follows: 10 min 95 °C followed by 40 cycles of 15 s for 95 °C and 1 m for 60 °C. The ∆CT values were performed from *ACTB* normalization, and further obtained the ∆∆CT values from control group. The relative expression rates were obtained from the 2^−∆∆CT^ algorithm. In terms of Western blot, cells were lysed by PRO-PREP^TM^, which was purchased from iNtRON Biotechnology (Gyeonggi-do, Korea), and incubated on ice for 30 min. Cells were centrifuged at 13,000 rpm for 15 min at 4 °C, and the supernatant was then quantified for SDS-polyacrylamide gel electrophoresis. Blots were blocked in 5% skimmed milk for 1 h and hybridized with primary antibodies of His-tag (Abcam, Cambridge, MA, USA), Collagen I (GeneTex, San Antonio, TX, USA), each at a ratio of 1:1000, and SOX2 (GeneTex, San Antonio, TX, USA), ACTB (Sigma, St. Louis, MO, USA), each at a ratio of 1:5000, for overnight hybridization. These data were confirmed with three independent experiments.

### 4.4. Promoter Construct and Assay

The promoter sequence of *COL1A1* was divided into 4 parts which included a consensus SOX2 binding site (T/A)TTGTT (−724~+3), an AP2 (−342~+3), an SP1 (−221~+3) binding sequence, and a TATA box only (−177~+3), as described in [23], and these fragments were linked to the luciferase reporter vector pGL3-basic (Promega, Madison, WI, USA). A SOX2del of *COL1A1* was conducted by removing the ATTGTT(−693~−687) in the promoter sequence, and cloned into the same vector. All plasmids were prepared and transfected by FuGENE HD^®^ Transfection Reagent (Promega). The subsequent reaction was catalyzed and measured by Steady-Glo^®^ Luciferase Assay System (Promega) and a luminance ELISA reader (Fluoroskan Ascent FL, Thermo Fisher Scientific, Cleveland, OH, USA), following the manufacturer’s instructions. The luciferase activity was obtained by three independent experiments, with six replicates for each group.

### 4.5. Chromatin Immunoprecipitation Assay

Total cell lysates (1.5 mg) were isolated from TGF-β-induced NHLF with an additional BP-treated group. The lysates were stored on ice, and sonication was performed with UP100H (Hielscher Ultrasonics GmbH, Teltow, Germany) to break into a 100–200 base pair length. The protein-DNA complexes (150 µg with protease inhibitor) were allowed to hybridize with SOX2 antibodies (10 µg Genetex) at 4 °C overnight. Protein A/G beads (Millipore, Hayward, CA, USA) were added and rotated at 4 °C for 2 h, and washed 4 times, then heated 65 °C to reverse cross links. Real-time PCR was performed to detect *COL1A1* promoter sequence. The internal control used an equal DNA concentration from the control cell lysate.

### 4.6. Bleomycin-Induced Pulmonary Fibrosis and Tissue Collection

Male C57BL/6 mice were purchased from the National Laboratory Animal Center and animal studies were approved by the China Medical University Institutional Animal Care and Use Committee. Four-week aged mice were housed in a clean enclosure and allowed to accommodate for a week; they had free access to a standard diet. Bleomycin (0.045 U dissolved in 50 µL PBS) or PBS was then injected with intratracheal instillation into the mice, allowing a recovery of 3 days. Bleomycin-treated mice were then randomly grouped into BP treatment (10, 50 mg/kg) and olive oil (in both vehicle and no bleomycin controls) orally every other day (*n* = 6 in all four groups), as illustrated in Figure 5A. On Day 30, animals were sacrificed to collect bronchoalveolar lavage fluid (BALF) and lung tissue. BALF collection is performed using cold PBS (0.5 mL), which was gently injected and pulled out 5 times through the trachea with a syringe, then stored on ice. The BALF samples were performed by magnetic bead-based multiplex immunoassays (Bio-Plex) (BIO-RAD Laboratories, Milano, Italy) following manufactures’ instructions. Lung tissues were removed for histopathology and protein extraction, and divided into formalin fixation and −80 °C frozen storage, respectively. Fixed tissues were embedded and followed by H&E or Masson’s trichrome stain. 

### 4.7. Pulmonary Function Test in Mice

Bleomycin–treated mice were administered BP orally with two dosages and olive oil as a vehicle control. Pulmonary function was measured by unrestrained Whole Body Plethysmography (WBP) (Buxco Electronics, Inc. Wilmington, NC, USA). The WBP detected atmospheric change to obtain data, such as respiratory rate and Tidal volume, in a specific time range. We assessed non-invasive airway responsive data using the WBP with a single-chamber prior to animal sacrifice, and as the chamber pressure changes, the box flow signal derived the following data: Inspiratory time (Ti); expiratory time (Te); relaxation time (TR), defined by the time for declining 36% of the total expiratory area; peak inspiratory flow (PIF) and peak expiratory flow (PEF); tidal volume (VT); accumulative volume (AV); end inspiratory pause (EIP); end expiratory pause (EEP); and respiratory rate (RR). The Penh (enhanced Pause) is referred to as an empirical parameter to limit airflow. Mice were incubated in the chamber to prevent light and noise, and the data collection period was set at 5~15 min to exclude the accommodation time.

### 4.8. Pathologic Morphology Staining and Evaluation

After lung tissues were obtained from sacrificed animals, samples were fixed in 3.7% formaldehyde for 2 d. The dehydration, clearance, and infiltration of all tissues were performed by a Histoprocessor (Tissue-Tek; Sakura, Tokyo, Japan). Paraffin embedded tissues were cut at a 4 μm serial section and stained with H&E. The procedure of Masson’s trichrome staining was previously described [16]. Briefly, sections were immersed in Bouin’s solution and then stained with Mayer’s hematoxylin solution, Biebrich scarlet–acid, phosphomolybdic acid–phosphotungstic acid, and aniline blue reagents (Sigma-Aldrich, Steinheim, Germany), respectively, with a ddH_2_O wash between each reagent. The samples were dried and mounted on glass slides, and sections were examined using a microscope (IX70; Olympus Tokyo, Japan). The Ashcroft scores were evaluated with blinded label and confirmed by a pathologist, who defined the assignment of grades from 0 to 8 [37]. The hydroxyl proline was examined with each group of lung tissues (*n* = 6), and equal weight of lung tissues was minced, following the manufacturer’s instructions of the assay kit (Cell Biolabs, San Diego, CA).

### 4.9. Statistical Analysis

All of the experiments were performed in three or more independent experiments. Statistical analysis of the results (*n* > 5) between groups was analyzed by ANOVA to obtain the *p*-value < 0.05, and a subsequent *post hoc* test with Dunnett’s test by SigmaPlot V12.5.

## Figures and Tables

**Table 1:** Parameters of respiratory function in BP-treated groups were compared with vehicle control in bleomycin-induced mice. Data represent the mean ± standard error of the mean of *n* = 6 mice per group. * denotes a significant decrease of *p* < 0.05 by ANOVA followed by *post hoc* analysis of Dunnett’s test.Data represent the mean standard error of the mean of *n* = 6 mice per group. * denotes a significant decrease of *p* < 0.05 by ANOVA and subsequent post hoc test with Dunnett’s test if the *p*-value below 0.01.

Variable	Normal	10 mg/kg	50 mg/kg	Vehicle
**Frequency (breaths/m)**	452.5 ± 2.8	447.4 ± 75.4	460.1 ± 27.1	393.1 ± 41
**Tidal volume (mL)**	0.046 ± 0.001	0.046 ± 0.002	0.046 ± 0.001	0.048 ± 0.002
**Accumulated volume (mL)**	247.23 ± 201.77	141.03 ± 99.02 *	288.96 ± 7.5 *	124.42 ± 48.14
**Minute volume (mL/m)**	20.51 ± 0.06	19.94 ± 2.62 *	20.11 ± 2.59 *	18.08 ± 1.36
**Inspiratory time (s)**	0.0569 ± 0.0023	0.0623 ± 0.0086	0.0587 ± 0.008	0.0587 ± 0.0037
**Expiratory time (s)**	0.092 ± 0.003	0.101 ± 0.029	0.096 ± 0.027	0.078 ± 0.041
**Peak inspiratory (mL/s)**	1.313 ± 0.008	1.271 ± 0.076	1.269 ± 0.139	1.361 ± 0.048
**Peak expiratory (mL/s)**	1.103 ± 0.008	1.046 ± 0.07	1.037 ± 0.066	0.848 ± 0.411
**Relaxation time (s)**	0.0598 ± 0.0043	0.0586 ± 0.0168 *	0.0501 ± 0.0106 *	0.0815 ± 0.0075
**End inspiratory pause (ms)**	5.514 ± 1.089	5.296 ± 0.916	4.955 ± 0.377	6.047 ± 1.293
**End expiratory pause (ms)**	7.1129 ± 2.339	21.35 ± 24.67	14.08 ± 9.7 *	37.9 ± 16.21
**Enhanced pause (Penh)**	0.62 ± 0.002	0.608 ± 0.068 *	0.582 ± 0.105 *	0.742 ± 0.091

## Abbreviations

Term	Definition
ECM	extracellular matrix
SOX2	sex determining region Y (SRY)-box 2
COL1	type I collagen
COL1A1	type I collagen α1
TGF-β	transforming growth factor beta
BP	butylidenephthalide
NHLF	normal lung fibroblast cell lines
E-cad	E-cadherin
i.t.	intratracheal
WBP	whole body plethysmography
BAL	bronchoalveolar lavage
